# Supplementary material for: Are carbon emissions trading and green financial instruments synergistic? -Comprehensive quantitative research based on content analysis
Source: PLoS One. 2024 Mar 7;19(3):e0298601. doi: 10.1371/journal.pone.0298601 (PMC10919601; doi:10.1371/journal.pone.0298601)
Supplement: S1 Data — (ZIP) [file pone.0298601.s001.zip › Data/地级市Z_F工作报告.zip!/╜¡╦╒/╬▐╬2╩╨/└┤╫╘╣1⁄2╓┌║┼í╛┬φ┐╦╩2╛▌═°í┐_2016.docx]

各位代表：

　　现在，我代表无锡市人民政府向大会报告工作，请予审议，并请市政协委员和其他列席人员提出意见。

　　2015年工作回顾及“十二五”发展成就

　　2015年是“十二五”规划的收官之年。面对复杂宏观经济形势和严峻挑战，在中共无锡市委的正确领导下，全市上下深入学习贯彻习近平总书记系列重要讲话特别是视察江苏时重要讲话精神，认真落实中央和省委、省政府决策部署，牢牢把握稳中求进总基调，积极适应经济发展新常态，统筹做好改革发展稳定各项工作，预计全市地区生产总值同比增长7.1%，一般公共预算收入增长8.1%，城镇和农村常住居民人均可支配收入分别增长8%、8.5%，居民消费价格涨幅控制在省定范围内，经济社会实现平稳健康发展。

　　(一)突出稳增长工作重点，经济运行稳中有进。坚持把稳增长作为紧要任务，全力促进经济运行稳定在合理区间。突出稳定工业经济。积极开展“稳心留根”和“暖企行动”，预计全市规模以上工业总产值和增加值分别增长1.8%、4.4%，规模以上工业企业利税和利润分别增长4.5%、5%。着力改善投资结构。加大产业投资力度，固定资产投资增长7%，其中工业投入增长13%，180个重大项目当年完成投资1000亿元。实际到位外资32亿美元，其中制造业占比达55%，三星SDI偏光板等60个超3000万美元项目成功落户。积极扩大消费需求。努力稳定传统商贸业，加快培育新的消费增长点，积极发展电子商务等新兴消费模式，社会消费品零售总额增长9%，电子商务交易额超3800亿元，增长18%。预计全市商品房成交面积和成交金额分别增长15%、18.5%。大力推动外贸发展。制定并落实外贸扶持政策和贸易便利化措施，对外贸易基本稳定;服务外包执行总额、离岸执行金额均增长30%。有效稳固金融支撑。成功举办省级金融机构支持无锡转型发展政银企合作恳谈会，预计社会融资规模新增1455亿元，其中新增本外币贷款496.3亿元，企业直接融资858.7亿元，同比分别增加135、32.5、334.8亿元;为1343家中小企业提供转贷应急资金126亿元;新增境内外上市公司6家、新三板挂牌企业67家。多措并举处置不良贷款248亿元、三年来累计处置不良贷款768亿元，银行业不良贷款余额和不良率同比实现“双下降”。加强政府性债务风险管控和存量债务置换，融资平均成本同比下降3个百分点左右。

　　(二)明确调结构主攻方向，转型升级步伐加快。确立并实施产业强市战略，加快打造以“四化”为引领，以新兴产业为先导、先进制造业为主体、现代服务业为支撑的现代产业发展新高地;加快构建以市场为导向、企业为主体、高校院所为支撑的产业科技创新体系，并制定出台促进现代产业发展的系列配套政策。增强科技创新驱动力。组织实施苏南国家自主创新示范区建设三年行动计划，加快推进国家传感网创新示范区建设，加强高层次人才引进和培育，预计全社会研发投入占地区生产总值比重达2.8%，全市科技进步贡献率达62%。积极推进产学研资用协同创新，累计建成国家级国际科技合作基地9家、省级以上工程技术研究中心502家，支持实施超级计算机等一批国家重大科技专项，华中科技大学无锡研究院、华进半导体封装技术研究所入选省产业技术研究院预备所。大力实施人才强企工程，推动开发园区引资与引智并举，一批民营企业与10位诺奖得主、11位外国院士牵手合作。高标准推进国家知识产权示范城市建设，万人有效发明专利拥有量25件，获第十七届中国专利奖5项。实施“创新之家”培育计划，开展“众创空间”建设六大行动和“智汇无锡”大学生创业系列活动，建成“众创空间”14家，新增大学生创业园10家。深入推进智慧城市建设，“一中心四平台”上线运行，成为全国首个高标准全光网城市，无锡(国家)智能交通产业园成立。推动先进制造业提质增效。大力发展“互联网+”经济，促进信息化和工业化深度融合，省级两化融合示范试点企业达249家。加快传统产业改造升级，深入实施“千企技改”工程，技改投入占工业投资比重达69%。积极推动企业兼并重组，长电科技等一批企业成功实现跨国并购。全面深化质量强市建设，支持企业品牌创建、标准创新，承担和参与制修订国内外标准93项，一汽锡柴荣获“全国质量奖”，阳光集团、兴澄特钢荣获“中国质量奖提名奖”。壮大新兴产业发展规模。新增高新技术企业353家，高新技术产业产值占规模以上工业总产值比重提高到41.5%，新兴产业产值增长10%。推进现代服务业提档升级。生产性服务业加快发展，新增省级制造业服务化示范企业10家。大力发展旅游业，预计实现旅游总收入增长10%，江南古运河、翠屏山和江阴徐霞客旅游度假区获批省级旅游度假区，无锡游客满意度全省第一。加快服务业集聚区建设，无锡食品科技园建设全面启动、人力资源服务产业园开园运营，苏南快递产业园成为全国首个国家级快递示范园区，无锡国家数字电影产业园实现销售增长170%。服务业增加值占地区生产总值比重达49.1%，提高0.7个百分点。提升农业现代化水平。扎实推进国家现代农业示范区建设，新增高标准农田1万亩、高效设施农业(渔业)2.75万亩，预计农业园区化比重提高到46%。江苏无锡国家农业科技园获批，洛社尚田农庄探索建设全省首家六次产业园。

　　(三)增强内生型发展动力，改革开放全面深化。坚持问题导向和需求导向，扎实抓好各项改革任务落实落地。深化行政审批制度改革。协同推进简政放权、放管结合、优化服务，向社会公布政府部门责任清单，完善行政权力事项清单管理办法，集中清理涉企服务收费。“一办三中心”政务服务平台在全省率先实现行政审批、为民服务、公共资源交易一体化规范运行。全面实施“三证合一”、“一照一码”登记制度，新登记内资企业和注册资本分别增长15.3%、46.5%。加强事中事后监管，建立健全“证照联动”监管和信用约束机制。深化经济体制改革。有序推进国资国企改革，加快国有资产证券化步伐，大力发展地方金融机构，国联证券在香港联交所成功上市，三房巷集团财务公司挂牌运作，北京银行、苏州银行、常熟农商行落户无锡，“苏民投”在锡筹备工作取得阶段性进展。积极推进滨湖区省级服务业综合改革试点。加快投融资体制改革，设立产业引导股权投资基金，推进南长滨河新城等一批PPP项目试点。

　　稳妥推进农村土地确权、农村产权流转交易市场建设和家庭农场认定登记工作，开展土地确权试点村比例超过70%，31个镇级产权流转交易服务中心建成运行，惠山区获准农村土地经营权抵押贷款试点。天然气价格、水价及其阶梯价格改革稳妥推进。全面完成不动产登记职责整合和机构组建任务。深化社会领域改革。深化社会事业“管办分离”改革取得突破。城市公立医院改革全面启动，医药价格综合改革平稳实施，区域医疗联合体试点取得实效，明慈心血管医院、百佳妇产医院等一批民营医院落地无锡，社会资本办医格局初步形成。扎实开展国家养老服务业综合改革，在全国地级市率先出台《养老机构条例》。进一步推进户籍制度改革，落实城乡户口统一登记和大市范围户口通迁制度，全面实施居住证制度。进一步扩大对外开放。全面对接上海自贸区试验政策，复制推广48项改革创新事项。加快开发区提档升级，成立中韩(无锡)科技金融服务合作区，成功创建出口“两车”质量安全示范区。加强口岸功能建设，进境食用水生动物、进口肉类指定口岸获国家批准，苏南硕放国际机场引进境外航空公司13家，旅客吞吐量增长10.3%。抓住国家“一带一路”战略机遇，加快“走出去”步伐，完成境外协议投资17.5亿美元，增长20%，柬埔寨西港特区建设取得新进展。成功举办第四届世界佛教论坛和第二届全球锡商大会，无锡灵山获批海峡两岸交流基地。

　　(四)夯实可持续发展基础，生态建设扎实推进。把生态文明建设作为可持续发展的重要战略，切实加强环境治理和保护，城乡生态环境质量持续改善。强化环境综合治理。持续加强太湖水污染防治，加快新沟河延伸拓浚等重点工程建设，加强入湖河道整治和饮用水源保护，积极开展蓝藻打捞处置，连续第八年实现太湖安全度夏。建立大气污染防治长效机制，组织开展污染源清单编制工作，推进实施1433个大气污染防治重点项目，PM2.5平均浓度较2013年下降18.7%。完成原锡钢地块污染土壤修复工程。加强生活垃圾收运处置体系建设管理，加快实施桃花山生活垃圾填埋场扩建一期续建工程，有序推进锡东垃圾焚烧发电厂复工前期工作，建成区生活垃圾机械化收集率、无害化处理率100%。强化环保执法监管，查处环境违法案件1003件，否决、劝退不符合环保要求项目48个，环保部专项督查反馈问题整改落实到位。推进节约集约发展。全面完成节能减排目标任务，预计万元GDP能耗下降5%以上，主要污染物排放总量削减完成省下达任务。无锡热电厂关停，蓝天燃机热电联产项目建成投运。深入实施“1236”节约集约用地战略，加大闲置土地处置力度，单位建设用地GDP产出预计达5.81亿元/平方公里，继续保持全省第一。加强生态环境保护。严格执行省生态红线区域保护规划，稳妥推进并落实生态补偿机制。深入推进“绿色无锡”建设，完成造林绿化1.33万亩，市区新增绿地500万平方米。加快宛山荡、贡湖湾等湿地公园建设，预计自然湿地保护率达44%。美丽乡村建设取得新成效，宜兴市洑西村被评为2015年中国最美休闲乡村，宜兴市白塔村、惠山区桃源村获评“江苏最美乡村”。无锡成为国家生态保护与建设示范区试点城市，获评“全国十佳生态文明城市”，再次荣膺中国十大宜居城市。

　　(五)强化惠民生发展理念，民生福祉不断增强。深入实施民生幸福工程，健全完善公共服务“六大体系”，12件80项为民办实事项目全面完成。完善城乡基础设施。加快太湖新城、锡东新城、惠山新城、马山国际旅游度假区等重点片区建设，完成古运河风光带核心段提升改造工程，西环线、北中路、广石路等一批城市道路建成通车，地铁1号线南延工程、3、4号线一期、苏锡常南部高速公路前期工作扎实推进。统筹实施老城区更新改造，整治改造棚户区(危旧房)、旧住宅区分别达57.7万、327万平方米，竣工拆迁安置房244.7万平方米。市区拆除违法建筑17.7万平方米，完成14条主要道路包装出新和42条背街小巷综合改造，增设1000余家苏邮便民服务点和快递示范门店。加大电网建设投入，中心城区、太湖新城高可靠性供电示范区建设加快推进。提升城区排水防涝能力，成功经受去年夏天超历史极值水位的考验。提高民生保障水平。实施积极的就业政策，全市新增城镇就业14.85万人，实现高校毕业生就业4.19万人，扶持自主创业1.15万人，帮扶就业困难人员再就业1.99万人。提高市区城镇最低生活保障和居民养老保险待遇标准，连续第11年增加企业退休人员养老金，市区3.58万人次获大病保险补助7193万元。组织实施全民参保登记，社会保障覆盖面进一步提高，“五险合一”公共服务标准化项目通过验收。完善养老服务设施建设，全市新增养老床位2190张，每千名老人拥有养老床位40张。残疾人托养服务能力不断提升。健全平价商店常态惠民机制，全市206家平价商店累计惠民金额超2.1亿元。积极推进公交优先和绿色出行，新辟、优化调整公交线路41条，新增插电式新能源公交车190辆，地铁1、2号线运行平稳，客流量逐步增加。推进教育事业协调发展，高标准新建、改扩建幼儿园35所，新增省四星级高中2所，无锡开放大学挂牌成立，入选全国首批现代学徒制试点城市。优化医疗服务体系结构，提档升级建设社区卫生服务中心和卫生院18家。加快创建国家公共文化服务体系示范区。成为全省首批公共体育服务体系示范区，成功申办2018年世界击剑锦标赛，成功举办无锡国际马拉松赛等一批大型国际赛事。加强和创新社会治理。建立社区事务准入机制，全市各街道(镇)全面推进“政社互动”，社会组织总数达12041个，顺利通过首批全国社区治理和服务创新实验区验收。加强信访工作，有效调处化解一批信访积案。着力维护公共安全，全年未发生重特大安全生产事故，连续14年实现事故起数、死亡人数“双下降”。深化平安无锡建设，社会治安形势总体稳定。加强食品药品监管，食品、药品安全检测合格率分别达98.15%、99.65%。成功创建全国文明城市。外事、史志、档案、对台、侨务、港澳事务、民族宗教、人武、人防、双拥、气象、防汛防台、防震减灾等工作取得新成绩，妇女、儿童、红十字、老龄、残疾人等事业有了新发展。

　　(六)加强服务型政府建设，依法行政效能提高。扎实开展“三严三实”专题教育活动，着力加快政府职能和作风转变，政府自身建设得到进一步加强。扎实推进法治政府建设。制定《无锡市法治政府建设规划(2015—2020年)》，出台《无锡市重大行政决策程序规定》，进一步完善市政府常务会议、市长办公会议等议事决策机制。提交地方性法规议案5件，出台市政府规章5件，全年受理行政复议566件。认真落实人大常委会审议、专题询问和执法检查意见、政协专题协商意见，5个政府部门向人大常委会报告工作并接受评议，积极回应人大代表、政协委员对社会热点问题的关注，办结议案、建议、提案674件。切实提升行政效能。建成投资项目在线审批监管平台，整合再造跨部门业务流程，行政审批(服务)事项承诺件提速率39.3%，群众办事更加便捷。12345政府公共服务热线期限内办结率98.9%，群众回访满意率98.4%。实施新一轮政府机构改革。工商、质监、食品药品监管体制改革落实到位，完成卫生计生、住房建设机构职责整合，市区部分行政区划调整工作积极稳妥推进。强化行政权力监督。认真整改落实省委巡视组反馈意见，严肃查处一批腐败案件。对领导干部经济责任审计进一步加强。严肃财经纪律，稳增长政策落实情况跟踪审计有力推进，财政预决算和“三公”经费信息全部公开。公务用车改革基本完成。

　　2015年经济社会发展目标任务基本完成，标志着“十二五”发展顺利收官。“十二五”时期，在国内外宏观经济环境严峻复杂、经济下行压力加大、困难和挑战明显增多的情况下，全市人民团结奋斗、攻坚克难，抢抓机遇、不懈努力，较好完成了“十二五”规划纲要确定的主要目标任务。

　　这五年，我们注重抓好发展第一要务，经济实力继续增强。预计全市地区生产总值由“十一五”末5793.3亿元增加到8500亿元、年均增长9.2%，常住人口人均GDP由9.2万元增加到13.1万元。一般公共预算收入由511.9亿元增加到830亿元、年均增长10.1%。三次产业结构由1.8：55.4：42.8调整到1.7：49.2：49.1。高新技术产业产值占规模以上工业总产值比重由34.2%提高到41.5%，新兴产业总产值由4103.7亿元增加到9800亿元、年均增长15.1%。全社会研发投入占GDP比重由2.5%提高到2.8%，高新技术企业由646家增加到1395家，科技进步贡献率由56.5%提高到62%。

　　这五年，我们注重激发体制机制活力，改革开放不断深化。全面推进66项重点领域和关键环节改革，深入实施“管办分离”改革、行政区划调整、政府机构改革、财税金融改革、公立医院和医药价格综合改革、农村产权制度改革等一批重大改革事项。境内外上市公司由65家增加到94家、新三板挂牌公司增加到104家，地方法人金融机构由12家增加到19家，经认定的总部性机构63个。货物贸易进出口总额由612.2亿美元增加到684.8亿美元、年均增长2.1%，其中出口总额由362.7亿美元增加到422.3亿美元、年均增长4%。到位注册外资171.7亿美元，占改革开放以来到位注册外资总和的34.5%。国际友城由34个增加到48个。

　　这五年，我们注重统筹协调发展，城乡建设稳步推进。全市常住人口由637.56万人增加到651万人，常住人口城市化率由71%提高到75%。京沪高铁、宁杭高铁无锡段建成通车，地铁1、2号线建成投运;新建一级以上公路98.7公里、市区城市道路508公里、改扩建农村公路585公里;市区新辟公交线路57条，调整公交线路199条，公交分担率由24.2%提高到28%。完成192个城市环境综合整治项目，9079个自然村全部完成环境整治，获评国家级美丽乡村2个、省级美丽乡村14个。

　　这五年，我们注重坚持环保优先方针，生态环境持续改善。预计万元GDP能耗下降25%，化学需氧量、氨氮、二氧化硫、氮氧化物四项主要污染物排放量分别削减20%、20%、21%、23%。太湖无锡水域水质稳定在Ⅳ类，饮用水源地水质100%达标。全市28.7%面积划为生态红线保护区。新增造林18.6万亩、城市绿地3706万平方米，预计全市林木覆盖率和自然湿地保护率分别由24.1%、24%提高到27%、44%。成为全国首个建成国家生态城市群的地级市。

　　这五年，我们注重树立为民惠民理念，民生水平逐步提高。预计城镇和农村常住居民人均可支配收入分别由27750元、14002元增加到45000元、24100元，年均分别增长10.5%、11.9%。新增城镇就业71万人。全市最低工资标准由960元增加到1630元;市区居民基础养老金、企业职工退休金、城乡低保标准分别由200元、1479元、420元增加到355元、2421元、700元，年均分别增长12.2%、10.4%、10.8%。预计城乡基本社会保险覆盖率提高到98%以上。完成棚户区(危旧房)改造150万平方米，旧住宅区综合整治改造957万平方米，竣工拆迁安置房2714.8万平方米、保障房61833套。

　　这五年，我们注重加强幸福无锡建设，社会事业全面发展。成为全国首批“义务教育发展基本均衡”城市，新建、改扩建幼儿园215所、小学88所、中学68所。成立江南大学无锡医学院，太湖学院成功转设独立设置的民办本科高校。新建、改扩建公立医院20家、社区卫生服务中心40家，新成立民办医院44家，全市医院床位数由1.98万张增加到3.16万张，养老床位数由2.37万张增加到4.69万张。大运河无锡段参与申报世界文化遗产成功，惠山祠堂群列入国家申遗目录，无锡灵山被确定为世界佛教论坛永久会址。环太湖国际公路自行车赛、斯诺克世界杯和无锡国际马拉松赛成为三大国际品牌赛事。

　　各位代表，过去一年和“十二五”取得的成绩来之不易，是党中央、国务院和省委、省政府正确领导和关怀支持的结果，是全市广大干部群众共同努力奋斗的结果。在此，我代表无锡市人民政府，向在各个领域和不同岗位上辛勤劳动、无私奉献的全体市民，向辛勤工作在村组、社区、企业等基层组织和单位的广大干部员工，向人大代表、政协委员，向各民主党派、工商联、各人民团体和各界人士，向中央及省在锡单位，向驻锡人民解放军、武警官兵和公安干警，向所有参与、支持和关心无锡建设与发展的海内外朋友，表示衷心的感谢，并致以崇高的敬意!

　　在肯定成绩的同时，我们也清醒地认识到面临的挑战和存在的不足，主要是：经济增长乏力，有的经济指标没有完成年度预期目标;转型升级任务艰巨，先进制造业和新兴产业对经济发展支撑不够有力;部分行业过剩产能消化缓慢，中小企业生产经营困难;财政收入增速放缓与刚性支出增加并存，基本公共服务供给相对不足;改革开放措施落地见效不快，体制机制障碍仍然存在;资源环境约束趋紧，生态环境问题依然比较突出;一些领域风险隐患尚未根本消除，维护经济社会稳定面临较大压力;服务型政府建设任重道远，机关工作作风和能力水平有待进一步转变和提高，反腐倡廉力度仍需加大，等等。我们将进一步强化忧患意识，正视矛盾、直面问题，在今后工作中努力加以解决。

　　“十三五”发展的总体要求

　　各位代表，根据《中共无锡市委关于制定无锡市国民经济和社会发展第十三个五年规划的建议》，市政府组织制定了《“十三五”规划纲要(草案)》，提请大会审议。

　　“十三五”时期是无锡建设经济强、百姓富、环境美、社会文明程度高新无锡的重要时期，是高水平全面建成小康社会的决胜阶段和积极探索开启基本实现现代化建设新征程的重要阶段。进入“十三五”，国内外形势发生深刻变化。世界经济在深度调整中曲折复苏，新一轮科技革命和产业变革蓄势待发，同时国际金融危机深层次影响仍长期存在，外部环境不确定因素增多。我国经济发展进入新常态，经济发展速度、发展方式、发展动力和经济结构调整呈现一系列新变化新特点，这为无锡经济转型升级提供了重要契机，也形成了倒逼压力。总的看，无锡发展仍处于可以大有作为的战略机遇期，但也面临诸多矛盾叠加、风险隐患增多的严峻挑战，经济下行压力加大，负重爬坡的阶段性特征非常明显。面对严峻复杂的经济形势，我们必须增强发展信心，这份信心来自于“十二五”发展打下的良好基础，来自于优越的投资环境、扎实的产业根基、活跃的民营经济和勤劳智慧的干部群众。我们将保持良好精神状态，充分把握有利条件机遇，克服不利因素影响，不自满、不懈怠、不松劲，努力把各项工作做得更好，为高水平全面建成小康社会而努力奋斗。

　　“十三五”发展的主要预期目标是：经济保持中高速增长、迈向中高端水平，提前实现地区生产总值和城乡居民人均收入比2010年翻一番。地区生产总值年均增长7.5%;高新技术产业产值占规模以上工业总产值比重达45%，服务业增加值占地区生产总值比重达50%以上;全社会研发投入占地区生产总值比重达3.1%;万元GDP能耗比“十二五”期末下降15%，主要污染物排放量在2015年基础上削减幅度达到国家和省的考核要求。

　　做好“十三五”工作，必须全面贯彻习近平总书记系列重要讲话特别是视察江苏时重要讲话精神。习近平总书记系列重要讲话特别是视察江苏时重要讲话精神，是无锡工作的根本遵循和行动指南。我们要以讲话精神统一思想行动、凝聚智慧力量，牢牢把握“四个全面”战略布局，紧紧围绕“两个率先”光荣使命，着力推动“五个迈上新台阶”，努力建设经济强、百姓富、环境美、社会文明程度高的新无锡，确保总书记重要讲话精神在无锡落地生根、开花结果。

　　做好“十三五”工作，必须自觉践行“五大发展理念”。中央“十三五”规划建议提出的创新、协调、绿色、开放、共享五大发展理念，是今后相当长时期我国发展思路、发展方向、发展着力点的集中体现。我们将注重用新的发展理念引领发展行动，形成更加鲜明的发展导向，着力在解决发展动力、发展不平衡、人与自然和谐、发展内外联动、社会公平正义等方面出实招、破难题、建机制，努力实现更高质量、更有效率、更加公平、更可持续的发展。

　　做好“十三五”工作，必须结合无锡实际积极实施“六大发展战略”。市委“十三五”规划建议从市情实际出发提出的重大战略，对适应新情况、指导新实践具有重要意义。着力实施创新驱动核心战略，使创新成为推动发展的主引擎;实施产业强市主导战略，全力打造现代产业发展新高地;实施全面开放战略，不断拓展开放新空间;实施新型城镇化和城乡发展一体化战略，促进区域在更高平台上实现均衡特色发展;实施可持续发展战略，加快建设资源节约型、环境友好型社会;实施民生共建共享战略，提高公共服务均等化水平，切实增强广大市民的获得感和幸福感。

　　做好“十三五”工作，必须不断提升无锡特色名城品质。得益于历史文化积淀、自然山水禀赋和勤劳智慧人民群众的接续努力，无锡作为历史文化名城、百年工商名城、旅游名城、智慧城市、宜居城市的特色品质，在国内外享有较高的知名度和认同感。我们要进一步提升和放大无锡的名城效应，彰显城市特色，擦亮城市品牌，不断提升城市美誉度和知名度。

　　2016年工作任务

　　今年是“十三五”开局之年，做好今年经济社会发展工作意义重大。今年政府工作的总体要求是：全面贯彻党的十八大和十八届二中、三中、四中、五中全会精神，以习近平总书记系列重要讲话特别是视察江苏时重要讲话精神为指引，认真落实中央和省委、省政府决策部署，紧紧围绕高水平全面建成小康社会和建设“强富美高”新无锡的总目标，按照“五位一体”总体布局和“四个全面”战略布局的要求，自觉践行创新、协调、绿色、开放、共享发展理念，全面实施创新驱动核心战略、产业强市主导战略、全面开放战略、新型城镇化和城乡发展一体化战略、可持续发展战略、民生共建共享战略，适应经济发展新常态，坚持稳中求进总基调，更加注重提高发展质量和效益，着力深化改革开放，着力稳定经济增长，着力推动转型升级，着力保障改善民生，着力防范化解风险，全力确保经济平稳健康发展和社会大局和谐稳定，努力实现“十三五”发展良好开局。

　　全市经济社会发展主要预期目标是：地区生产总值增长7.5%左右;一般公共预算收入增长7.5%左右;城镇登记失业率控制在3.5%以内;居民消费价格涨幅控制在3%以内;万元GDP能耗和主要污染物排放削减量完成省下达目标任务。上述主要预期目标，考虑到“十三五”开局之年的工作要求，兼顾了新常态下保持适当增长速度的需要，又为深化改革和调整结构腾出空间。

　　经济的稳定增长是做好全年工作的基础和前提。面对宏观环境的变化，必须把稳增长作为重点工作抓紧抓好。充分发挥投资的关键作用，以重大项目建设为抓手积极扩大有效投资，增加公共产品供给，提高投资质量和效益。制定扩大政府投资实施意见，加快推进一批事关长远发展的基础设施项目建设，确保基础设施投资有较大增长。全年继续安排市级重点项目180个，年内完成投资超1000亿元，固定资产投资增长10%左右。充分发挥消费的基础作用，多措并举挖掘传统消费潜力，积极培育信息、文化、教育、健康、养老等消费新业态，巩固和提高消费对经济增长的贡献率，努力实现社会消费品零售总额增长10%左右。充分发挥出口的支撑作用，巩固传统优势市场，积极开拓新兴市场，千方百计稳定外贸进出口，努力扭转出口下滑态势，力争出口实现正增长。加大金融服务实体经济力度，积极拓宽融资渠道，有效增加产业贷款，努力实现社会融资规模1600亿元，其中新增本外币贷款800亿元。大力推动企业直接融资，争取新增境内外上市公司6家、总数突破100家。

　　在稳增长的基础上，今年重点抓好以下工作：

　　(一)坚持产业强市导向，着力打造现代产业发展新高地

　　坚持以“智能化、绿色化、服务化、高端化”为引领，加快推动产业转型升级。充分保护和激发各类企业增加投入、兴办实业的热情，做到对外资企业、引进企业、本土企业一视同仁，对大中型企业、小微企业一视同仁，对国有企业、民营企业、混合所有制企业一视同仁，为各类市场主体创造机会均等、公平竞争的发展环境。

　　加快发展先进制造业、新兴产业和现代服务业。突出先进制造业主体地位，扎实开展中国制造2025无锡行动，重点发展“七大先进制造业”，制定推进计划，明确工作重点，开展主题招商活动，抓好目标任务落实。聚焦高端装备制造、智能制造、整机制造和关键零部件制造，努力在新能源汽车等整机制造上实现新突破。加快推进工业化和信息化深度融合，不断提高现代产业智能化水平。建立“千企技改”长效机制，加大传统产业改造支持力度。大力弘扬“工匠精神”，加快培育一批“专精特新”中小企业。争取规模以上工业增加值增长7.5%左右。推动新兴产业集群式发展，充分发挥已有产业优势，做强做大集成电路、物联网、节能环保、新能源、新材料、生物医药、信息技术、智能电网、通用航空等新兴产业，培育一批千亿级产业集群，争取实现新兴产业产值增长10%以上。推进军民融合式发展，支持帮助符合条件的民营企业进入国防科技工业领域。推动现代服务业乘势而上，加快发展生产性服务业，大力发展跨境贸易、现代金融、研发设计、服务外包、物流仓储、商务中介和总部经济，优化提升传统商贸业态，争取实现服务业增加值增长8%以上。深入实施质量和品牌战略，开展质量标准提升行动，不断提高质量强市建设水平。用足用好国家支持实体经济发展的政策和我市支持现代产业发展的配套政策，加强政策宣传解读，优化政策申报程序，尽可能让企业早享受、快享受、多享受政策。

　　坚持招商引资和“稳心留根”并重。强力开展产业招商引资活动，全力引进50亿元甚至100亿元的重大产业项目。加快实施一批重大技改项目，确保实现工业投入增长12%以上。加强利用外资工作，争取到位注册外资34亿美元，其中制造业项目利用外资占比60%左右。广泛深入开展本土企业、现有企业“稳心留根”活动，特别是对制造业中小企业给予更多支持和帮助。树立“好企业”正确导向，只要产品有市场、企业有利润、员工有收入、政府有税收、符合环保要求，就要为企业生存和发展留下空间，提供充分的土地、规划、信贷、政府服务等保障。强化各级高新区、开发区、特色园区发展现代产业主阵地作用，加强科学考核，优化政策机制，努力实现高于地区平均水平的产业增长。

　　推进农业现代化建设。培育新型农业经营主体，促进一二三产业融合发展。提高农业物质装备和技术水平，新增高标准农田1万亩、高效设施农业(渔业)2万亩。提升农业科技创新能力，大力发展生物农业、智慧农业、生态农业。加强区域粮食安全保障体系建设。推进供销社综合改革，加快发展农民专业合作社和为农服务示范社，提升农村合作经济水平。做好第三次全国农业普查工作。

　　(二)突出创新驱动战略，着力构建产业科技创新体系

　　把创新作为引领发展的第一动力，积极推进大众创业万众创新，无论是企业的创新、高校院所的创新、还是人才团队的创新，都给予大力支持;无论是关键技术攻关、企业技术改造，还是小发明小革新，都给予积极鼓励，切实为实体经济插上科技创新的翅膀。

　　大力推进两个国家创新示范区建设。推进苏南国家自主创新示范区建设，落实“三年行动计划”，在“一区三核多特”全面推广落实中关村科技创新优惠政策，使之成为科技创新重要增长极。推进国家传感网创新示范区建设，组织开展关键技术攻关，联合国家行业主管部门建设物联网创新示范基地，加快物联网技术和产品应用推广，支持推动物联网龙头企业做大做强，争取实现物联网产业营业收入增长20%左右。

　　深入推进政产学研用合作。强化企业在科技创新中的主体地位，支持鼓励企业采取跨国收购、兼并重组方式引进先进技术，支持鼓励企业与高校院所开展成果转化合作，支持鼓励企业通过引进人才开展自主研发。积极推进地方政府、骨干企业与高校院所合作建立高水平研发机构，深化与清华大学、华中科技大学、中科院微电所等的战略合作，加强共性技术平台、检测检验和实验平台建设，为企业创新提供有力支持。大力实施“太湖人才”计划，继续推进人才强企工程，完善落实相关扶持服务政策，引进和培养科技领军人才、高技能领军人才和高水平创新团队，打造优秀企业家队伍。积极发挥“欧美同学会”报国无锡基地作用，为海外人才搭建回国创业桥梁。充分利用现有科研办公用房载体，采取鼓励政策招引科技型企业和研发机构入驻，打造一批功能完善、服务高效的“众创空间”。加强知识产权保护，严厉打击侵权行为。争取全社会研发投入占地区生产总值比重达到2.82%，万人有效发明专利拥有量达到27件。

　　加快推进科技金融发展。支持和鼓励科技型企业到中小板、创业板上市，以及在新三板、各地股权交易中心挂牌，争取新增新三板挂牌公司40家。大力发展新型科技金融机构，创新科技金融产品和服务，积极开展股权、知识产权等质押贷款，争取累计发放科技风险补偿贷款50亿元，增长10%以上。充分发挥产业引导股权投资基金导向作用，设立一批新兴产业基金、天使投资基金，规范发展P2P、股权众筹等支持创新创业的互联网金融模式，促进投资机构和社会资本更多投向科技创新企业。

　　(三)落实供给侧改革要求，着力增强改革开放新动力

　　坚持以深化改革促进扩大开放，以扩大开放促进深化改革，尊重基层首创精神，鼓励基层先行先试，让一切劳动、知识、技术、管理、资本的活力竞相迸发，让一切创造社会财富的源泉充分涌流。

　　推动落实供给侧结构性改革五大任务。一是去产能。注重运用市场机制、经济手段、法治办法化解过剩产能，加大政策引导力度，完善企业退出机制，因地因企制宜，消化一批、转移一批、整合一批和淘汰一批过剩产能，同时坚决遏制新的过剩产能盲目扩张。二是去库存。注重打通供需通道，消化房地产库存，稳定房地产市场。积极推行棚户区(危旧房)改造、经济适用房保障对象货币化安置和补贴。探索形成购租并举的住房制度，把公租房扩大到非户籍人口，满足新市民住房需求。鼓励投资购买库存商品房，发展以住房租赁为主营业务的专业化企业。三是去杠杆。注重提高企业权益资本比重、降低负债率，主动释放信用违约风险。有效化解地方政府债务风险，加大存量债务置换力度，严控新增政府性债务规模。进一步加强金融风险化解处置，争取实现银行业不良贷款继续“双下降”。扎实做好防范和处置非法集资工作，严格控制设立各类投资理财类公司，有效遏制非法集资蔓延势头。四是降成本。注重打好“组合拳”，大刀阔斧帮助企业降低成本。规范涉企收费行为，落实结构性减税和定向减税政策，构建普惠性政策扶持体系。实施稳岗补贴政策，切实降低企业用工成本。五是补短板。注重保持有效投资力度，扩大有效供给，支持企业技术改造和设备更新，培育发展新产业，加强软硬基础设施建设，夯实打牢农业基础。

　　继续推进全面深化改革。全面落实国家和省下放取消的行政审批事项，全面落实行政权力清单管理制度，探索开展相对集中行政许可权改革。规范清理行政审批中介服务，建立政务服务网上中介超市。全面实施不动产统一登记，继续深化商事登记制度改革，加强事中事后监管。优化投资项目建设管理流程，完善在线审批监管平台，形成审批联动监管机制。整合建立统一的公共资源交易平台，构建法治化、规范化、透明化运行机制。深化投融资体制改革，积极实施财政资金“拨改投、拨改贷、拨改转”，加快发展PPP融资模式。深化国资国企改革，完善法人治理结构，健全薪酬管理和激励机制，推进国有资产证券化，加快发展混合所有制经济。大力发展地方法人金融机构，支持红豆集团发起设立锡商银行，加快组建“苏民投”、消费金融公司和金融租赁公司，推动无锡农商行上市。全面深化资源性产品、公用事业和公益性服务等重点领域价格改革。深化农村产权制度改革，完成土地承包经营权确权颁证工作，健全农村产权流转交易市场。统筹推进教育、医疗、养老、文化、社会治理等重点领域改革，狠抓各项改革任务落实。深入实施国家养老服务业综合改革、省级“两型社会”综合配套改革和滨湖区服务业综合改革试点。

　　继续推进高水平对外开放。主动服务和融入国家开放大局，认真组织实施我市参与国家“一带一路”和长江经济带发展战略三年行动计划，更多借鉴复制上海自贸区成功经验。大力推动开发园区、综合保税区转型升级，争取江阴临港经济开发区、惠山经济开发区升级为国家级经济技术开发区，江阴保税物流中心升级为国家级综合保税区。大力推进中韩(无锡)科技金融服务合作区、中法(无锡)智慧城市先行示范基地建设，争取设立更多的中外合作园区和示范区。加快航空物流园建设。进一步扩大内河港口和机场开放效能，加快推进跨境贸易电子商务、进口肉类指定口岸、药品进口口岸、国际邮件互换局建设。鼓励更多企业“走出去”，支持办好柬埔寨西港特区。大力推进城市国际化进程，建设一批国际化的教育、医疗、体育和文化服务机构，努力打造国际化和谐人居环境。

　　(四)加强城市建设管理，着力推进区域协调发展

　　扎实推进新型城镇化，推动产城融合发展，提升城市管理内涵，强化“一体两翼”基础设施互联互通，促进城乡发展一体化。加强历史文化保护，彰显国家历史文化名城底蕴。

　　提升城市建设和管理水平。适应于行政区划调整，积极实施梁溪区、新吴区资源整合，优化功能和产业布局，推进太湖广场北侧中信大厦和南丰汇等项目建设。大力发展楼宇经济，着力盘活一批闲置商业楼盘，促进主城区商业繁荣。积极开展老城更新改造，继续实施背街小巷综合改造和主要道路包装出新。推进城市精细化管理，完善数字化城管建设，争创省优秀管理城市。完善太湖新城、锡东新城、惠山新城等重点区域功能，加快产业和人口导入。进一步完善综合交通体系。增强苏南硕放国际机场服务能力，实现旅客吞吐量500万人次、货邮吞吐量10万吨。推进无锡火车站南广场综合改造工程。开工建设地铁3号线一期、1号线南延工程，扎实做好地铁4号线一期前期工作。推进蠡湖大道、江海高架西延等一批城市道路快速化改造，打通钱皋路、环山东路、桐桥港路等一批“断头路”。优化公交线网布局，新辟调整公交线路10条以上。完善城市配套设施，改造自来水管网60公里、自来水水表出户改造4000户，新增天然气用户5万户，新建污水管网14公里。加快“世界一流电网”建设，推广智能用电小区建设和水电气一体化采集，建成电动汽车充电桩1500个以上。加强海绵城市建设，提升防洪减灾能力。

　　大力推进锡澄宜一体化。全力做好锡澄、锡宜重大交通基础设施建设对上争取工作，启动实施一批重大项目，扎实推进南沿江铁路、盐泰锡常宜铁路、常宜高速、宜长高速公路建设前期工作，开工建设苏锡常南部高速公路，加快新锡澄路等重点道路建设，推进跨市(县)、区道路沟通。实施锡澄运河市区段整治工程，启动建设宜兴丁蜀通用机场，开展江阴通用机场前期工作。充分发挥江阴、宜兴区位和资源优势，增强副中心城市功能，支持江阴建设国际化现代化滨江名城、增强宜兴对苏浙皖交界地区的辐射功能。

　　促进城乡统筹协调发展。强化城市规划引领，做好城市总体规划修编研究工作，完善城乡一体化发展规划，推动城镇建设、土地利用、产业发展、生态建设等规划有机融合，促进“多规合一”。落实大市范围内户口通迁制度，稳妥有序推进农业转移人口市民化。推动公共财力向农村倾斜，提升城乡公共服务均等化水平。优化城镇规模结构，高标准推进一批重点镇和特色镇建设，改善城镇整体面貌。完善现代农业生态涵养和农村文化传承功能，促进古镇、古村落保护和发展，打造一批具有江南水乡特色的美丽乡村。

　　加强旅游名城建设。加强旅游资源整合，完善旅游基础设施和配套服务体系，改善城市旅游环境。加快旅游休闲集聚区发展，推进旅游景区、度假区转型升级，充分利用新媒体提升旅游营销水平。加强旅游与乡土风情、山水资源、文化创意和休闲产业等融合，加快推进万达文化旅游城等一批重点旅游项目建设，塑造旅游城市品牌形象，着力打造国际知名、国内一流的休闲度假旅游目的地。

　　加快智慧城市建设。积极实施“互联网+”行动计划和大数据战略，加速城市大数据中心数据汇聚、分析和应用，完成智慧城市运营体验中心建设。充分发挥云计算、物联网、移动互联网等新一代信息技术在城市管理、经济发展和民生服务领域的支撑作用。加快实施智慧政务、交通、健康、社区等重点提升工程。实现光纤宽带在全市住宅小区、乡镇农村全覆盖。扩大政府数据公开规模，探索政府数据与社会数据的大融合。

　　(五)搞好生态文明建设，着力保护青山绿水环境

　　顺应人民群众对良好生态环境的热切期盼，切实加强生态文明建设，积极推进绿色发展，着力抓好群众反映强烈的水污染、大气污染、黑臭河道、酸雨等突出环境问题的整治，努力建设天蓝地绿水清的美丽无锡。

　　加强环境综合整治。落实国务院《水污染防治行动计划》，开展新一轮太湖治理，完成2家工业园区、13家污水处理厂整改或提标，92家涉水企业提标、整治或淘汰搬迁;完成20块排水达标区创建和30%已建成区复查;关闭禁养区养殖场80家，整治适养区小型养殖场445家、规模畜禽养殖场313家;对38条河道实施综合整治，重点治理前胡村浜、创业河等6条黑臭河道。全面施行《大气污染防治法》，完善大气质量监测预警体系，将细颗粒物等环境指标列入约束性指标，确保PM2.5平均浓度降幅达到国家考核要求，空气质量达标天数比例好于上年。严格执行全市煤炭总量控制方案，建成西区燃气热电项目，加快建设东亚电力(无锡)燃气电厂一期项目。继续实施燃煤小锅炉整治，强化工业废气、机动车尾气、建筑扬尘等污染治理，加强秸秆综合利用。加大新能源汽车推广应用力度。扩大生活垃圾分类收集试点，启动餐厨垃圾处理设施建设，完成桃花山垃圾填埋场技改工程和气体资源化利用项目，推进垃圾焚烧飞灰填埋场规划建设，争取群众支持锡东垃圾焚烧发电厂复工。深化关停搬迁工业企业原址场地的环境管理，积极开展工业企业退出场地土壤修复试点。推进城乡美化绿化，新增绿化面积1万亩。

　　加大生态保护力度。严格落实主体功能区计划，出台生态红线区域保护规划。加强资源节约集约利用，实施“百万吨节能量工程”和五大节能改造行动计划。坚持最严格的节约用地制度，切实保护耕地和基本农田，盘活存量土地，加强低效用地再开发。强化节能降耗刚性调控，加快推进清洁生产、循环经济和资源综合利用。严格执行环境准入和污染物排放标准，完成年度节能减排任务。完善主要污染物排污权有偿使用和交易试点制度，继续推行环境污染责任保险，完善生态补偿制度，深入推进水环境区域补偿。探索领导干部自然资源资产离任审计试点。加强环境监管执法队伍建设，依法从严打击环境违法行为。倡导市民合理消费和绿色生活，推动形成勤俭节约的社会风尚。

　　(六)践行共享发展理念，着力提高人民生活水平

　　全面落实惠民举措，围绕推进基本公共服务均等化，积极实施一批重大民生工程，扎实办好10件80项民生实事，做好项目推进工作，加强每月跟踪监测，努力为人民群众多办实事、好事，使全体市民在共建共享发展中有更多获得感。

　　积极促进就业创业。坚持就业优先战略，新增城镇就业8万人以上。鼓励企业稳定用工，实施就业质量提升工程，着力解决结构性就业矛盾。做好高校毕业生等重点群体就业工作，积极开发公益性岗位，帮助困难就业人员就业。实施技能人才振兴工程，提升农村转移劳动力素质，新增高技能人才1.5万人，办好省第三届技能状元大赛。鼓励大众创新创业，年内扶持自主创业1万人。积极构建和谐劳资关系。多渠道增加居民收入，完善工资收入分配制度，继续提高低保家庭人均收入，确保居民收入增长与经济增长同步。

　　完善社会保障体系。积极推进全民参保，重点解决好本地户籍人员断保问题，实现企业职工养老、医疗保险分别扩面5万人以上，失业、工伤和生育保险分别扩面3万人以上。强化社保欠费清缴联动机制，完善基础养老金合理调整机制，推进机关事业单位养老保险制度改革，完善城乡统一的居民医疗保险制度。继续实施“阳光医保工程”，加强医保支出管理。建立健全现代工伤保险制度。有效应对人口老龄化挑战，提高居家、社区、机构养老服务水平，新增养老床位2000张，城乡社区居家养老机构基本实现全覆盖，为2.5万户特定困难老人家庭提供居家养老援助服务。积极用好国家棚户区改造政策，推进棚户区(危旧房)改造30万平方米，整治旧住宅区185万平方米、城中村60个。加强经适房、廉租房、拆迁安置房项目续建和物业长效管理，有序推进农村住房改造。帮助1500户以上各类住房保障对象解决住房困难。

　　繁荣发展教卫文体事业。加快发展教育事业。新建、改扩建幼儿园30所，新增学额5000个。推进义务教育学校标准化建设，培育建设25所“新优质学校”，深化普通高中课程基地建设。加快推进太湖新城国际学校、和畅小学、信成小学和市少年宫新宫建设。提升教师队伍素质，实施乡村教师支持计划。重视做好外来务工人员随迁子女义务教育工作。加快发展高等教育，扩大本科层次办学规模，支持太湖学院争取通过教育部本科教学水平评估。完善现代职业教育体系，实施现代学徒制试点。健全民办教育扶持政策，支持和规范民办教育发展。加快发展医疗卫生事业。进一步深化公立医院改革，完善覆盖城乡的基本医疗卫生制度。实施改善医疗服务行动计划，积极开展预约和分级诊疗服务。加快推进江南大学无锡医学院和附属医院建设，强化医学人才培养和专(学)科建设。引进国内外优质医疗资源，完善多元办医格局，推进医疗养老融合发展。推动中医药事业加快发展。提高人口出生素质，实施全面两孩政策。加快发展文体事业。探索建立公共文化服务标准体系，完成市文化馆新馆和非遗展示馆改造工程，扩大公共文化设施免费开放范围，开展各类公益文化活动，争创国家公共文化服务体系示范区。推进惠山祠堂群、荡口古镇申报世界文化遗产，加强锡绣、吴歌等非物质文化遗产保护传承和开发利用，振兴惠山泥人、宜兴均陶等传统手工艺。贯彻全民健身国家战略，积极发展青少年校园足球，争创第二批国家级科学健身示范区。精心办好环太湖国际公路自行车赛、国际马拉松赛、亚洲击剑锦标赛等重大体育赛事。

　　切实做好扶贫帮困工作。全面落实社会救助制度，努力实现对困难群众的精准救助。高度重视残疾人救助扶持，不断拓展社会化助残新领域。加大对经济薄弱村帮扶力度，启动实施新一轮帮扶工作，坚持走开发式扶贫之路，积极盘活集体资产，促进联合开发经营，增强经济薄弱村“造血”功能，实现全市经济薄弱村脱贫转化30个以上。继续做好南北挂钩和对口支援工作。

　　加强社会治理创新。巩固全国文明城市创建成果，加强对广大市民尤其是青少年的社会主义核心价值观教育，进一步提升城市文明程度。深化城乡社区“五化”治理，推进政社互动、“三社”联动，严格社区事务准入，切实减轻基层负担。促进基层社会治理精细化，鼓励和引导社会组织参与社会事务管理。健全群众利益表达、协调和保护机制，加强和改进信访工作，有效预防化解矛盾纠纷。落实安全生产责任制，加强市民安全意识教育，提高网络与信息安全保障水平。健全应急管理机制。加强放心消费环境建设，完善社会信用体系，创建社会信用体系建设示范城市。强化食品药品监管，实施食品安全行动计划，食品抽检率达到3批次/千人以上。深化平安无锡建设，严密防范、依法惩治违法犯罪活动，完善立体化现代化社会治安防控体系，完成主城区530万平方米老小区技防设施建设。做好民族、宗教等工作，发挥工会、共青团、妇联等群众团体作用。增强市民国防意识，推进“双拥”共建活动，提高优抚安置水平。

　　(七)遵循“三严三实”要求，着力加强政府自身建设

　　围绕“为民、务实、清廉”和“忠诚、干净、担当”的要求，切实加强和改进政府工作，不断创新管理方式，提升服务效能，为经济社会发展提供坚强保障。

　　全面依法行政。增强各级行政机关工作人员法律意识，严格按照法定权限和程序行使权力、履行职责。健全依法科学民主决策机制，完善政府法律顾问制度，加强和改进行政复议、行政应诉工作。深入推进政务公开，及时向社会公开经济社会发展重大决策。认真落实市人大及其常委会决议决定，自觉接受人大法律监督和政协民主监督，坚持重大事项向人大报告和政协通报，主动听取民主党派、人民团体和社会各界意见建议，自觉接受司法监督、新闻舆论监督和社会公众监督。

　　持续改进作风。深入落实领导干部挂钩联系工作机制，创新工作思路，健全协调服务机制，更多深入基层帮助解决实际困难，特别是要把为企业服务作为天职，以企业需求为导向，以企业满意为标准，让企业办事更方便、更快捷、更舒心，为企业营造优质的发展环境。坚决反对做表面文章、搞形式主义，大力弘扬主动作为、勇于担当精神，增强不进则退、无功即过意识，坚决克服不作为、慢作为和“慵懒散”现象。大力弘扬“定了干、马上办、办到底”精神，深入开展执行力监督检查，切实打通工作落实“中梗阻”。始终保持反“四风”高压态势，严格执行厉行节约、反对浪费各项规定，加强对部门预算与支出的规范管理，严格控制“三公”经费。

　　强化廉政建设。完善惩治和预防腐败体系，全面落实“一岗双责”，严格执行廉政规定，着力打造风清气正的政府工作队伍。加强对权力运行的监督制约，严肃纠正重点领域不正之风，完善12345政府公共服务热线，加强政风面对面平台建设，推动基层站所公共服务标准化全覆盖，不断提升政风行风建设水平。强化监察工作，健全行政执法监督机制。加强审计监督，对公共资金、国有资产、国有资源和领导干部履行经济责任情况实行审计全覆盖。

　　各位代表，潮起海天阔，扬帆正当时。让我们紧密团结在以习近平同志为核心的党中央周围，在中共无锡市委的坚强领导下，解放思想、凝心聚力，克难奋进、扎实工作，为高水平全面建成小康社会、开创“十三五”发展新局面、建设“强富美高”新无锡而努力奋斗!
